# Use of Vegetable Waste as a Culture Medium Ingredient Improves the Antimicrobial and Immunomodulatory Activities of *Lactiplantibacillus plantarum* WiKim0125 Isolated from Kimchi

**DOI:** 10.4014/jmb.2210.10049

**Published:** 2022-12-02

**Authors:** Seul-Gi Jeong, Ho Myeong Kim, Moeun Lee, Jung Eun Yang, Hae Woong Park

**Affiliations:** Technology Innovation Research Division, World Institute of Kimchi, Gwangju 61755, Republic of Korea

**Keywords:** Kimchi, lactic acid bacteria, *Lactiplantibacillus plantarum* WiKim0125, antimicrobial effect, anti-inflammation

## Abstract

Lactic acid bacteria (LAB) isolated from kimchi (a traditional Korean dish typically made of fermented cabbage) can provide various health benefits, including anti-obesity, antioxidant, anti-inflammatory, anticancer, and antimicrobial effects. In this study, we examined the antimicrobial and immunomodulatory effects of *Lactiplantibacillus plantarum* WiKim0125 cultured in de Man, Rogosa, and Sharpe (MRS) medium containing vegetable waste. Live bacterial cells were eliminated via supernatant filtration or heat treatment. The cell-free supernatant (CFS) obtained from culture broth containing kimchi cabbage waste (KCW), cabbage waste (CW), or onion waste (OW) showed significantly higher antimicrobial activity against skin pathogens (*Propionibacterium acnes* and *Staphylococcus aureus*) and foodborne pathogens (*Escherichia coli* and *Salmonella typhimurium*), with inhibition zones ranging between 4.4 and 8.5 mm, compared to that in conventional MRS medium (4.0–7.3 mm). In lipopolysaccharide-stimulated RAW264.7 cells, both supernatant and heat-inactivated *Lb. plantarum* WiKim0125 from culture media containing KCW and CW suppressed the production of inflammatory cytokines (72.8% and 49.6%, respectively) and nitric oxide (62.2% and 66.7%, respectively) without affecting cell viability. These results indicate that vegetable waste can potentially increase the antimicrobial and immunoregulatory potency of LAB while presenting a molecular basis for applying postbiotics to health products.

## Introduction

Recently, the health benefits of fermented vegetables have been of great public interest due to low coronavirus disease 2019 (COVID-19) death rates in Eastern Asia, Central Europe, sub-Saharan Africa, and the Middle East reportedly related to the habitual consumption of fermented vegetables [1, 2]. In particular, recent studies on the correlation between antioxidant transcription factors and COVID-19 found that the intake of kimchi, a representative Korean fermented dish, could alleviate COVID-19 symptoms by activating nuclear factor-erythroid 2-related factor-2 [3, 4]. Other health benefits of kimchi, such as anticancer, anti-obesity, anti-diabetes, and antioxidant effects, have also been well established, and are closely associated with lactic acid bacteria (LAB) and LAB-derived bioactive substances [5-11].

Although several genera of LAB have been developed as functional probiotics, safety concerns regarding the consumption of probiotics have been raised due to the possibility of side effects caused by the unexpected activity of live microorganisms [12, 13]. To prevent these risks, research into the beneficial effects of dead microbial probiotic cells, cell compounds, and metabolites has been conducted [14-16]. Jo *et al*. [17] reported that heat-killed *Lactobacillus curvatus* WiKim38 isolated from kimchi has immunoregulatory activity in dendritic cells and mice. In another study on kimchi LAB, both the cytoplasmic fraction and culture medium extract of *Weissella koreensis* OK1-6 inhibited intracellular lipid accumulation in adipocyte cells, resulting in an anti-obesity effect [18]. However, most research thus far has focused on live bacteria.

Using agricultural waste as a component of culture media has emerged as a circular economy strategy to cope with enormous amounts of waste. This waste can provide nutrients, including the organic carbon necessary for the growth of microorganisms [19, 20]. Agro-industrial by-product wastes from fruit, brewing, and dairy industries have been investigated for their ability to improve the biological activity of LAB and produce bioactive compounds [21-23]. Despite the many studies conducted for developing alternative culture media, the application of vegetable waste to kimchi LAB fermentation has not yet been studied.

In our laboratory, LAB isolated from kimchi were cultivated in de Man, Rogosa, and Sharpe (MRS) medium and screened for antimicrobial activity against pathogenic microorganisms. Of particular interest is *Lactiplantibacillus plantarum* WiKim0125, a rod-shaped, gram-positive, and facultative anaerobic bacteria, which has been donated to the Korean Culture Center of Microorganisms. Our objective in this study was to investigate the potential antimicrobial and anti-inflammatory properties of heat-inactivated *Lb. plantarum* WiKim0125 and its cell-free supernatant (CFS) in vitro. Following its characterization, the effect of vegetable waste-containing culture medium on improving biological activities was also comprehensively evaluated.

## Materials and Methods

### Bacterial Strain

*Lb. plantarum* WiKim0125 (KFCC 11871P) isolated from cabbage kimchi was obtained from the Eco-Friendly Process Technology Research Group Culture Collection at the World Institute of Kimchi (Gwangju, Korea). Stock cultures were stored at –80°C in MRS broth (BD Difco, USA) containing 15% glycerol. Working cultures were streaked onto MRS agar (BD Difco), incubated at 30°C for 24 h, and stored at 4°C.

### Culture Medium Formulation

The vegetable wastes used were kimchi cabbage waste (KCW), cabbage waste (CW), and onion waste (OW) from the respective counties of Haenam, Jindo, and Muan, located in South Jeolla Province, Korea. All agricultural wastes were dried for 5 days using a freeze dryer (FDT-8632; Operon, Korea) at –80°C, and then mechanically crushed into particles by an air jet mill (JM-LB; Kmtech, Icheon, Gyeonggi Province, Korea) and stored at –20°C until subsequent use. The freeze-dried wastes were added individually to MRS broth, accounting for 2% of the total volume. Lb. Plantarum WiKim0125 was cultured in MRS broth and the three different culture media at 30°C for 24 h.

### Microbial and Chemical Analysis of Culture Broth

The microbial populations, pH, and lactic acid content of the culture broth were measured via plating on MRS agar, a pH meter (Orion Star A211; Thermo Fisher Scientific, USA), and a high-performance liquid chromatography apparatus (Waters e2695; Waters Co., USA) at 50°C using an ion-exclusion column (Aminex HPX-87H; Bio-Rad, USA; elution was conducted with 0.05 N sulfuric acid at a flow rate of 0.6 ml/min and a wavelength of 210 nm), respectively.

### Preparation of CFS and Heat-Inactivated Bacteria

**Fig. 1:**
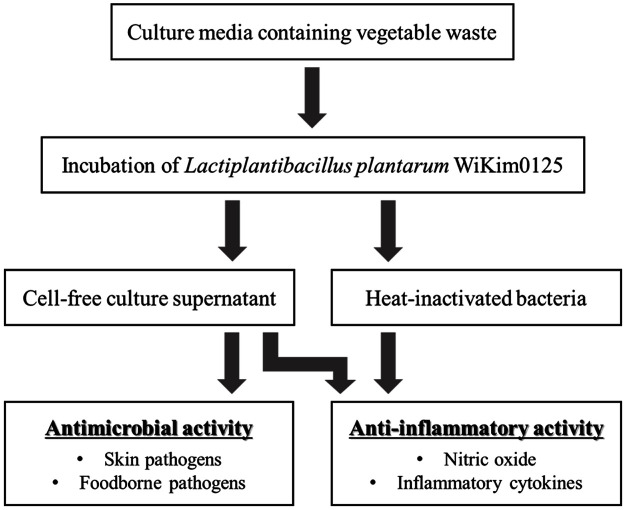
Experimental design for the in vitro analysis of the antimicrobial and immunoregulatory effects of cell-free supernatants of and heat-inactivated *Lactiplantibacillus plantarum* WiKim0125.

The culture broths were centrifuged at 4,000 ×*g* for 20 min at 4°C to separate the supernatant and precipitated cells. To obtain the CFS and heat-inactivated bacteria, the supernatant and precipitated cells were filtered with a sterile syringe filter (0.2 μm; Whatman, UK) and autoclaved for 15 min, respectively. The absence of LAB in the samples was confirmed via MRS agar plating and immediately followed by antimicrobial and anti-inflammatory assays. The experimental design is illustrated in Fig. 1.

### Antimicrobial Assay

The antimicrobial activity was evaluated as the inhibition zone size of indicator microorganisms following the agar diffusion method described by Balouiri *et al*. [24], with minor adjustments. Cultures of the following bacteria were used in this study: (i) skin pathogens *Propionibacterium acnes* (ATCC 6919) and *Staphylococcus aureus* (ATCC 12692) in reinforced clostridial medium (BD Difco) and tryptic soy broth (TSB; BD Difco), respectively; and (ii) foodborne pathogens *Escherichia coli* (ATCC 8739) and *Salmonella* Typhimurium (ATCC 13311) in TSB. For each microorganism, 1 ml was serially diluted in 9 ml of sterile phosphate-buffered saline (3M, USA), corresponding to approximately 10^8^ CFU/ml (0.5 McFarland standard), and then 0.1 ml of the appropriate diluent was spread-plated onto each corresponding medium. A total of 10 μl of the CFS was dispensed onto each plate with a diameter of 5 mm, dried for 30 min inside a biosafety hood with the fan running, and then incubated at 37°C for 24 h. The diameter of the inhibition growth zone was measured in mm using a digimatic caliper (CD-15CPX; Mitutoyo, Japan).

### Cell Culture

RAW264.7 macrophage cells (ATCC TIB-71) derived from mice were obtained from the Korean Cell Line Bank (Korea) and grown in Dulbecco’s modified Eagle medium (Gibco, USA) containing 10% fetal bovine serum (Gibco) and 1% penicillin/streptomycin (Gibco) in a humidified atmosphere with 5% CO_2_ at 37°C. The anti-inflammatory activity was evaluated in vitro using macrophage cells between the third and seventh passages.

### Cell Viability Assay

Macrophage cells were seeded at a density of 3 × 10^4^ cells/well in 96-well plates, incubated with CFS or heat-inactivated *Lb. plantarum* WiKim0125 for 24 h, and then washed with Dulbecco’s phosphate-buffered saline (Gibco). A total of 100 μl of dye solution containing XTT sodium salt (Sigma-Aldrich) and phenazine methosulfate (Sigma-Aldrich) was added to each well. The 96-well plates were incubated at 37°C in a 5% CO_2_ incubator for 2 h, and the absorbance was measured at 450 nm using a microplate reader (Infinite 200 Pro; Tecan, Switzerland). The cytotoxic effect of all the tested samples was determined as a percentage of the absorbance relative to that of untreated cells.

### Inflammatory Cytokine Measurement

RAW264.7 cells (3 × 10^6^ cells/well) were cultured with CFS in 6-well plates with 1 μg/ml lipopolysaccharide (LPS; Sigma-Aldrich, USA). After 24 h of incubation at 37°C in a 5% CO_2_ incubator, total RNA was extracted using TRIzol reagent and then reverse transcribed and amplified using a one-step quantitative real-time PCR (qPCR) kit (Qiagen, Germany). The qPCR reaction was conducted in a 25 μl reaction mixture containing 1 μl of template RNA, 2 μl of each primer (Table 1), 0.25 μl of reverse transcriptase mix, 12.5 μl of SYBR Green master mix, and 9.25 μl of RNase-free water. Thermal cycling was performed in a qPCR detection system (CFX Connect; Bio-Rad) with reverse transcription at 50°C for 10 min and enzyme activation at 95°C for 5 min, followed by 40 cycles of denaturation at 95°C for 10 s and annealing and extension at 60°C for 30 s. The result was normalized to that of β-actin.

### Nitric Oxide Measurement

RAW264.7 cells (3 × 10^4^ cells/well) were treated with heat-inactivated *Lb. plantarum* WiKim0125 in 96-well plates and stimulated with 1 μg/ml LPS at 37°C in a 5% CO_2_ incubator for 24 h. The cell culture supernatants were mixed with Griess reagent (Promega, USA) at a 1:1 ratio, and the mixture was placed in the dark for 15 min at 24 ± 2°C. The absorbance at 540 nm was measured using a microplate reader (Infinite 200 Pro; Tecan), and the amount of nitric oxide was calculated via a standard curve plotted against various concentrations of sodium nitrite (Promega).

### Statistical Analysis

All experiments were carried out three times with duplicate samples. Statistical analysis was performed via one-way analysis of variance using the Statistical Package for the Social Sciences (version 20; IBM Corp., USA). Significant differences between treatments were determined using Duncan’s multiple range test and set at *p* < 0.05.

## Results and Discussion

### Changes in Microbial Growth and Chemical Composition of Different Culture Broths

According to their efficacy and purpose, alternative culture media are divided into two groups: media for microbial growth and media for producing microbial compounds [20]. To better determine which group our vegetable waste-containing culture media fall into, the results of the microbial and chemical analyses of the culture broth containing KCW, CW, or OW of *Lb. plantarum* WiKim0125 are presented in Table 2. No significant differences were observed in the number of bacterial cells when grown in MRS or vegetable waste-containing media (*p* > 0.05). This shows that the alternative medium did not stimulate the growth of *Lb. plantarum* WiKim0125. In contrast, the production of lactic acid, generally thought of as the major metabolite of *Lactobacillus* species, significantly increased in KCW-, CW-, and OW-containing media compared with that in the MRS medium (*p* < 0.05). Although these differences had no significant effect on pH values, the increase in lactic acid production shows that these agricultural wastes are an appropriate substrate for lactic acid fermentation. Similar results have been observed with apple bagasse, rice bran, and mango peels [22, 25, 26]. The high glucose and fructose content in KCW, CW, and OW could be utilized as a carbon source and may explain the increased lactic acid production. Other compounds, including monosaccharides, disaccharides, oligosaccharides, malic acid, and citric acid, could also be metabolized by LAB [22, 27-29].

### Antimicrobial Effects of Culture Medium Containing Vegetable Waste

**Fig. 2:**
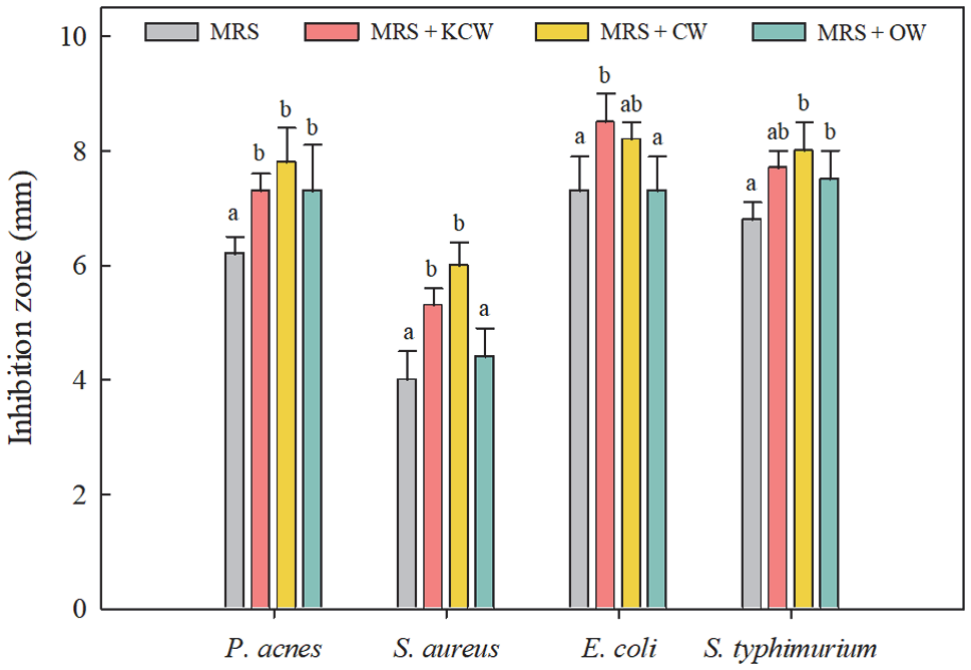
Antimicrobial effect of cell-free culture supernatant of *Lactiplantibacillus plantarum* WiKim0125 in MRS medium containing vegetable waste against *Propionibacterium acnes*, *Staphylococcus aureus*, *Escherichia coli*, and *Salmonella* Typhimurium. Error bars indicate standard deviations calculated from triplicate values. Values for the same pathogen followed by different letters are significantly different (*p* < 0.05). KCW, kimchi cabbage waste; CW, cabbage waste; OW, onion waste.

Fig. 2 shows the antimicrobial activity of the CFS of *Lb. plantarum* WiKim0125 cultured in vegetable waste-containing culture media and MRS medium against skin and foodborne pathogens. *E. coli* was the most sensitive to the CFS, with inhibition zones ranging between 7.3 and 8.5 mm, followed by *Salmonella* Typhimurium (6.8–8.0 mm), *P. acnes* (6.2–7.8 mm), and *S. aureus* (4.0–6.0 mm). The CFS of *Lb. plantarum* WiKim0125 showed an antimicrobial activity in all tested media; however, a higher degree of inhibition of pathogenic strains was obtained using the CFS formulated with agro-industrial wastes than with MRS broth. In particular, the CFS of *Lb. plantarum* WiKim0125 grown in KCW- and CW-containing media reduced the growth of indicator microorganisms by up to 50.0% more than those cultured in the MRS media. Our results concur with those of Linares-Morales *et al*.[30] and Leães *et al*. [31], indicating that food industry wastes, including apple bagasse, fish waste, and soybean meal, could be employed as cost-effective substrates for improving antimicrobial activity. The influence of agricultural waste-containing culture media on the antimicrobial effect of LAB corresponded to increased lactic acid production compared to that of the MRS medium (Table 2). Besides lactic acid, bacteria can produce other antimicrobial products, such as antimicrobial peptides. In the LAB proteolytic system, protein hydrolysate obtained from food industry by-products can allow the production of antimicrobial peptides [32, 33].

### Effect of Vegetable Waste-Containing Culture Media on Cell Viability

**Fig. 3:**
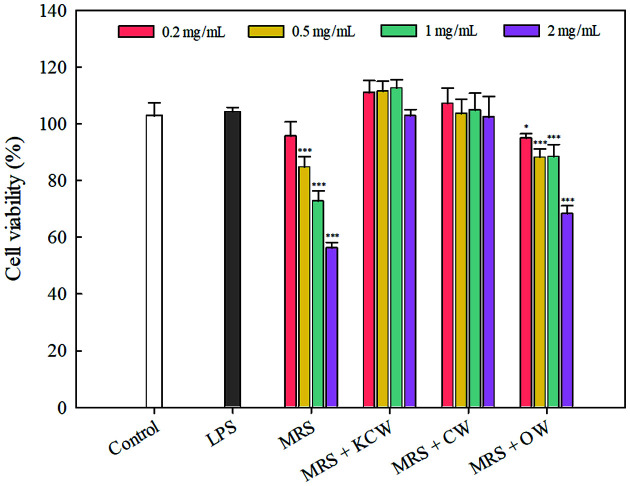
Cytotoxic activity of heat-inactivated *Lactiplantibacillus plantarum* WiKim0125 grown in MRS medium containing vegetable waste. Error bars indicate standard deviations calculated from triplicate values. *p < 0.05, **p < 0.01, and ***p < 0.001 indicate significant differences from the control. KCW, kimchi cabbage waste; CW, cabbage waste; OW, onion waste.

The cytotoxicity of CFS and heat-inactivated *Lb. plantarum* WiKim0125 grown in MRS and vegetable waste-containing culture media against RAW264.7 cells was examined using the XTT assay. No significant difference in cell viability between untreated and CFS-treated cells at concentrations of up to 3% was observed in all tested media (*p* > 0.05; data not shown). The low cytotoxicity of fermented food products with LAB has been previously reported for vegetable juice and purple sweet potato [34, 35]. As shown in Fig. 3, the viability of RAW264.7 cells dose-dependently decreased in the presence of different amounts of heat-inactivated *Lb. plantarum* WiKim0125 grown in each of the culture media. This result is consistent with that of a previous study, which concluded that heat-inactivated LAB strains decreased cell viability in a dose-dependent manner [35, 36]. Among the different culture media, KCW- and CW-containing media retained significantly higher cell viability than MRS and OW-containing media (*p* < 0.05). At 2 mg/ml, the viability of cells treated with heat-inactivated bacteria from KCW-and CW-containing media was 102.78 and 102.37%, respectively; however, cells from MRS and OW-containing media exhibited 56.17 and 68.33% viability, respectively. In the present study, heat-inactivated *Lb. plantarum* WiKim0125 cultured in KCW- and CW-containing media showed no cytotoxicity at concentrations up to 2 mg/ml.

### Effect of Vegetable Waste-Containing Culture Media on Anti-inflammatory Activity

**Fig. 4:**
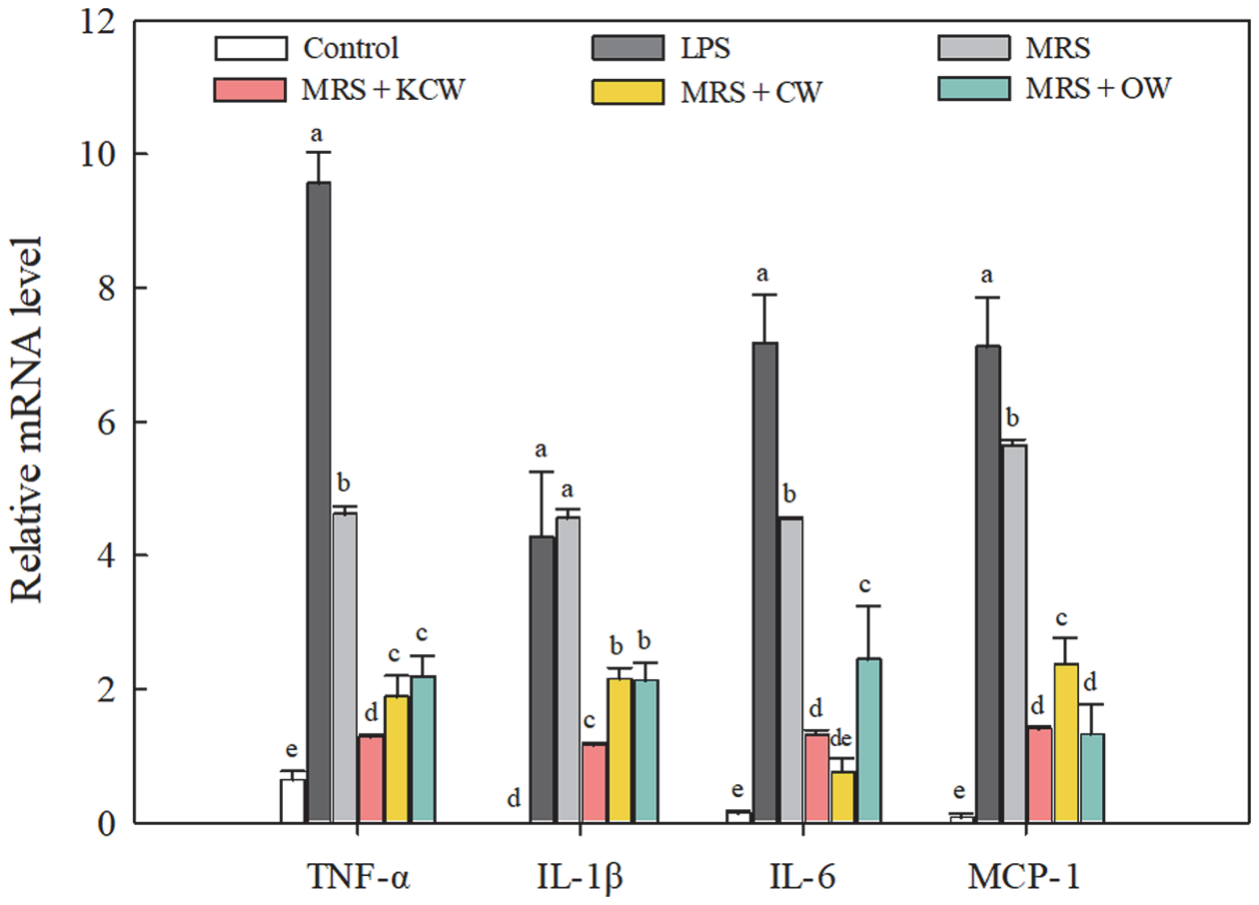
Effect of cell-free culture supernatant of *Lactiplantibacillus plantarum* WiKim0125 in MRS medium containing vegetable waste on cytokine production by LPS-induced RAW264.7 cells. Error bars indicate standard deviations calculated from triplicate values. Values for the same cytokine followed by different letters are significantly different (*p* < 0.05). KCW, kimchi cabbage waste; CW, cabbage waste; OW, onion waste; TNF-α, tumor necrosis factor-α; IL-1β, interleukin-1β; IL-6, interleukin-6; MCP-1, monocyte chemoattractant protein-1; LPS, lipopolysaccharide.

The relative mRNA expression levels of inflammation-related biomarkers, such as tumor necrosis factor-α (TNF-α), interleukin-1β (IL-1β), interleukin-6 (IL-6), and monocyte chemoattractant protein-1 (MCP-1), are shown in Fig. 4. These cytokines are secreted by macrophages in response to cell damage resulting from infection and are associated with immune response activation [37, 38]. In this study, cytokine production was higher in the LPS group than in the non-treated control group, and the treatment with CFS of *Lb. plantarum* WiKim0125 at 3% significantly suppressed LPS-induced cytokine production (*p* < 0.05). The immunomodulatory effects of various kimchi LAB strains, including *Lactococcus lactis* WiKim0124, *Companilactobacillus allii* WiKim39, *Lb. plantarum* LB5, and *Lb. brevis* G-101, have previously been proposed [35, 39, 40]. The anti-inflammatory effects of CFS from MRS-cultured *Lb. plantarum* WiKim0125 were significantly lower than those from the three vegetable waste-containing culture media (*p* < 0.05). In particular, the CFS of *Lb. plantarum* WiKim0124 from KCW-containing medium induced the lowest mRNA expression of all tested biomarkers, except for IL-6; it showed the greatest inhibitory effect on TNF-α expression, with a decrease of 86.6% compared to the LPS-alone treatment group. Moreover, mRNA expression levels of IL-1β, IL-6, and MCP-1 were also significantly decreased (*p* < 0.05), with the highest inhibitory effect on IL-6, with an 81.7% decrease compared to the LPS-alone treatment group.

**Fig. 5:**
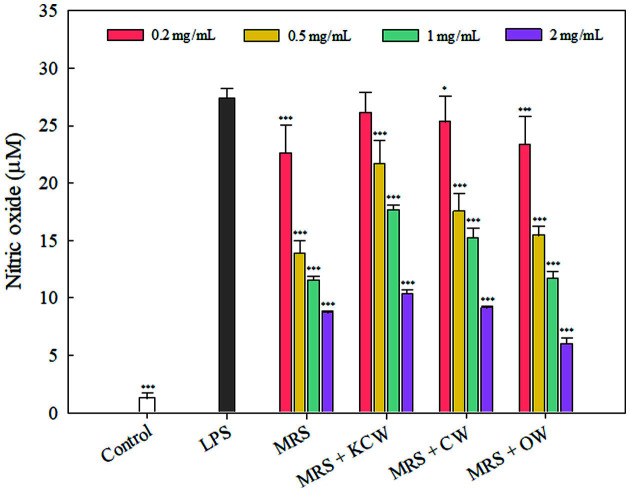
Effect of heat-inactivated *Lactiplantibacillus plantarum* WiKim0125 grown in MRS medium containing vegetable waste on nitric oxide production by LPS-induced RAW264.7 cells. Error bars indicate standard deviations calculated from triplicate values. *p < 0.05, **p < 0.01, and ***p < 0.001 indicate significant differences from the group treated with LPS alone. LPS, lipopolysaccharide; KCW, kimchi cabbage waste; CW, cabbage waste; OW, onion waste.

Similar to the results from the cytokine experiments, treatments with heat-inactivated *Lb. plantarum* WiKim0125 significantly suppressed LPS-induced NO production in a dose-dependent manner (Fig. 5, *p* < 0.05). In previous studies, and in agreement with our results, treatment with dead kimchi LAB showed an inhibitory effect on LPS-induced NO production [36, 41]. Although heat-inactivated *Lb. plantarum* WiKim0125 in OW-containing medium was associated with the lowest NO production at 2 mg/ml, heat-inactivated *Lb. plantarum* WiKim0125 in KCW- and CW-containing media also reduced NO production by 10.34 and 9.11 μM, respectively. Overall, based on cell cytotoxicity and NO production results, KCW and CW were appropriate for improving the immunoregulatory activity of heat-inactivated Lb. Plantarum WiKim0125. In addition, the polyphenol components of kimchi cabbage and cabbage inhibit oxidative stress and reduce cytokine expression [42]. Before this study, little information existed on the anti-inflammatory effect of culture media formulated with vegetable wastes, despite much earlier research on the antimicrobial effects. Although the mechanism has not been proven definitively, the increased anti-inflammatory activity of LAB could be attributed to the physiological components of agricultural waste, thereby increasing various LAB activities during fermentation. Yun *et al*. [35] suggested that components of vegetable juice, such as indol-3-carbinol, anthocyanins, and nitrate, increased immunomodulatory effects when fermented with kimchi LAB.

The present study suggests that culture media formulated with vegetable wastes can be effectively used to enhance the antimicrobial and anti-inflammatory activities of *Lb. plantarum* WiKim0125 isolated from kimchi. KCW and CW were the substrates that significantly induced the biological activities of both CFS and heat-inactivated *Lb. plantarum* WiKim0125; however, the active components should be identified by analyzing the soluble factors in the CFS and ligands on the heat-inactivated *Lb. plantarum* WiKim0125. Further studies are also necessary to investigate the mechanism by which vegetable waste-containing media improve biological activities. With a thorough understanding of this, use of agro-industrial wastes could be a potential strategy for enhancing the antimicrobial and immunomodulatory effects of kimchi LAB when used as dietary supplements or food ingredients.

## Figures and Tables

**Table 1:** Quantitative real-time PCR primer sequences.

Gene^a^	Forward primer	Reverse primer
β-actin	AGCCATGTACGTAGCCATCC	TTAAGCCATGCTCTGCAATG
TNF-α	ACGGCATGGATCTCAAAGAC	AGATAGCAAATCGGCTGACG
IL-1β	GCAACTGTTCCTGAACTCAACT	TCTTTTGGGGTCCGTCAACT
IL-6	CCTTCCTACCCCAATTTCCAA	AGATGAATTGGATGGTCTTGGTC
MCP-1	CCCCAAGAAGGAATGGGTCC	GGTTGTGGAAAAGGTAGTGG

^a^TNF-α, tumor necrosis factor-α; IL-1β, interleukin-1β; IL-6, interleukin-6; MCP-1, monocyte chemoattractant protein-1.

**Table 2:** Changes in the microbial population, pH, and lactic acid content of *Lactiplantibacillus plantarum* WiKim0125 grown in vegetable waste-containing culture media and MRS media^1^.

Media type^2^	Microbial populations (log CFU/ml)	pH	Lactic acid (g/l)
MRS	9.44 ± 0.50 ^a^	3.87 ± 0.03 ^a^	17.69 ± 0.04 ^c^
MRS + KCW	9.56 ± 0.27 ^a^	3.88 ± 0.03 ^a^	20.59 ± 0.31 ^b^
MRS + CW	9.52 ± 0.36 ^a^	3.85 ± 0.05 ^a^	20.41 ± 0.06 ^b^
MRS + OW	9.46 ± 0.43 ^a^	3.93 ± 0.07 ^a^	21.90 ± 0.16 ^a^

^1^Mean ± standard deviations of three replicates are shown. Values in the same column followed by the same letters (a,b,c) are not significantly different (*p* > 0.05).

^2^MRS, de Man, Rogosa, and Sharpe medium; KCW, kimchi cabbage waste; CW, cabbage waste; OW, onion waste.
